# Breastfeeding and early infection in the aetiology of childhood leukaemia in Down syndrome

**DOI:** 10.1038/sj.bjc.6605244

**Published:** 2009-08-25

**Authors:** J Flores-Lujano, M L Perez-Saldivar, E M Fuentes-Pananá, C Gorodezky, R Bernaldez-Rios, M A Del Campo-Martinez, A Martinez-Avalos, A Medina-Sanson, R Paredes-Aguilera, J De Diego-Flores Chapa, V Bolea-Murga, M C Rodriguez-Zepeda, R Rivera-Luna, M A Palomo-Colli, L Romero-Guzman, P Perez-Vera, M Alvarado-Ibarra, F Salamanca-Gómez, A Fajardo-Gutierrez, J M Mejía-Aranguré

**Affiliations:** 1 Unidad de Investigacion en Epidemiologia Clinica, Hospital de Pediatria, Centro Medico Nacional Siglo XXI (IMSS), Quetzal 27, Las Arboledas, 52950 Mexico DF, Mexico; 2 Unidad de Investigacion Medica en Enfermedades Infecciosas y Parasitarias, Hospital de Pediatria, CMN SXXI (IMSS), Las Arboledas, 52950 Mexico DF, Mexico; 3 Inmunogenetica, Instituto de Diagnostico y Referencia Epidemiologica, Mexico DF, Mexico; 4 Servicio de Hematologia, Hospital de Pediatria, CMN SXXI (IMSS), Las Arboledas, 52950 Mexico DF, Mexico; 5 Servicio de Hematologia Pediatrica, Hospital General ‘Dr. Gaudencio Garza’, Centro Medico Nacional ‘La Raza’ (IMSS), Mexico DF, Mexico; 6 Servicio de Oncologia-Hematologia, Instituto Nacional de Pediatria (INP), Mexico DF, Mexico; 7 Servicio de Onco-Hematologia, Hospital Infantil de Mexico Federico Gomez, Mexico DF, Mexico; 8 Servicio de Hematologia, Centro Medico Nacional ‘20 de Noviembre’ (ISSSTE), Mexico DF, Mexico; 9 Servicio de Hematologia, Hospital General de Mexico, Mexico DF, Mexico; 10 Subdirector de Hemato/Oncologia, Instituto Nacional de Pediatria (INP), Mexico DF, Mexico; 11 Departamento de Genetica, Instituto Nacional de Pediatria, Mexico DF, Mexico; 12 Genetica, Hospital de Pediatra, Centro Medico Nacional Siglo XXI (IMSS), Las Arboledas, 52950 Mexico DF, México

**Keywords:** leukaemia, Down syndrome, breastfeeding, infections

## Abstract

**Background:**

For a child to develop acute leukaemia (AL), environmental exposure may not be sufficient: interaction with a susceptibility factor to the disease, such as Down syndrome (DS), may also be necessary. We assessed whether breastfeeding and early infection were associated with the risk of developing AL in children with DS.

**Methods:**

Children with DS in Mexico City, and either with or without AL, were the cases (*N*=57) and controls (*N*=218), respectively. Population was divided in children with AL and with acute lymphoblastic leukaemia (ALL) and also in children ⩽6 and >6 years old.

**Results:**

Breastfeeding and early infections showed moderate (but not significant) association for AL, whereas hospitalisation by infection during the first year of life increased the risk: odds ratios (confidence interval 95%) were 0.84 (0.43–1.61), 1.70 (0.82–3.52); and 3.57 (1.59–8.05), respectively. A similar result was obtained when only ALL was analysed.

**Conclusion:**

We found that breastfeeding was a protective factor for developing AL and ALL, and during the first year of life, infections requiring hospitalisation were related to a risk for developing the disease in those children with DS >6 years of age. These data do not support the Greaves's hypothesis of early infection being protective for developing ALL.

Worldwide, acute leukaemia (AL) is the commonest type of childhood malignancy with an annual incidence of 30–50 cases per million children (Curado *et al*, 2007); Mexico City has one of the highest rates, at 58.4 cases per million (Mejia-Arangure *et al*, 2005a). Among the identified risk factors, individuals with Down syndrome (DS) below 20 years of age have 10- to 20-fold greater risk of developing AL compared with the general population (Robison, 1992; Ross *et al*, 2005).

According to the Greaves' hypotheses, late contact with infectious agents may lead to an abnormal or aberrant immune response that favors the development of acute lymphoblastic leukaemia (ALL) (Greaves, 2005). Correspondingly, early infections have a protective effect in ALL by promoting the physiological maturation of the immune system, whereas late infections promote an excessive proliferation of the lymphocytes (McNally and Eden, 2004; Menegaux *et al*, 2004; Greaves, 2006; O'Connor and Boneva, 2007).

Children with DS, due to their increased susceptibility to AL, provide a natural and valuable model for evaluating the interaction between the susceptibility for AL and the environment. In this study, we assessed whether breastfeeding and early infection were associated with the risk of developing AL in children with DS. Mother's milk is an important modulator of the immune response in infants because, while by providing the child with antibodies and immune cells, it can also be a source of transmission of infectious agents (MacArthur *et al*, 2008).

## Materials and methods

A case–control study was performed. All cases of DS and AL under 19 years of age diagnosed with AL during the period 1998–2006 were included in this study. These institutions treat children under this age and the major number of cases of ALL occurred during this age (Hasle *et al*, 2000). They were drawn from the six participating public institutions that treat children with cancer in Mexico City, under the auspices of the Instituto Mexicano de Seguro Social (IMSS), Instituto de Seguridad Social al Servicio de los Trabajadores del Estado (ISSSTE) and Secretaría de Salud (SS). Each diagnosis of AL was confirmed by means of a bone marrow aspirate and clinical study of karyotype.

Children with DS are enrolled in two types of institution in Mexico City. The Centres of Multiple Attention (CMA) and Specialized Centres that provide special education exclusively to children with DS. The controls for this study were drawn from three of those Specialized Centres: (1) John Langdon Down Institute, (2) Centro de Terapia Educativa CTDUCA and (3) Centro de Educacion Down Asociacion Civil CEDAC, where the karyotype is a requisite for admission of a child. Of such children under 19 years of age in the above-mentioned Specialized Centres, 78% were included in this study. These centres accept children from any part of Mexico City and if any child develops AL, care would be provided by one of the hospitals serving as sources of the cases. It should be noted that, although there are 76 other CMA in Mexico City that provide special care for children with diverse disabilities, including DS, none of these children were included in this study because their karyotypes had not been documented.

To collect the information required for the questionnaire (QMOD website; National Cancer Institute, 1998) for both groups, nurses went to the hospitals and institutions of special education, after obtaining a letter of informed consent from the parents. Parents who agreed to participate were then interviewed individually about child's medical history: birth weight of the child; breastfeeding; infections during the first year of life of the child (upper respiratory tract infections, bronchopneumonia, pneumonias, gastrointestinal infections, etc.); infections that required hospitalisation during the first year of life; antecedent of cardiovascular disease in the child; family history of cancer; personal habits of the parents, such as smoking and alcohol consumption; and age of the mother at delivery and socio-demographic aspects. The stacking level was used as a proxy for socio-economic status, and it was calculated according to the number of persons per room in a household. Classification, using the criteria of Bronfman *et al* (1988) was as follows: not crowded (up to 1.5), semi crowded (between 1.6 and 3.5) and crowded (3.6 and more). Low socio-economic level included both semi crowded and crowded.

### Statistical analyses

All the leukaemias were included in the analysis. The odds ratio (OR) and the 95% confidence intervals (CIs) were calculated in measuring the relation between breastfeeding, early infections, and hospitalisation due to infections and the control variables and AL in children with DS. Each of the independent variables (breastfeeding, early infections and hospitalisation due to infections) was stratified for each one of the control variables in order to control for confounding factors that may modify the effect. The ORs were estimated using an unconditional logistic regression analysis, controlling for the sex and age of the child, birth weight of the child, firstborn child, standard of living and cardiovascular diseases. A logistic regression model was also applied to the group of children with ALL. The statistical package used for this analysis was SPSS version 16.0 (2008 SPSS Inc., Chicago, IL, USA).

## Results

Of the 57 cases (1998–2006) of children with DS and leukaemia, 45 (79. 2%) had ALL; the remainder having acute myeloblastic leukaemia (Table 1). The average age of the cases at diagnosis of AL, and of the controls, at the time of interview with the parents, were 106 and 82 months, respectively (*P*=0.04). Of the 57 AL cases, 50 were aged 0–14 years, as were 39 of the 45 ALL cases. The birth weight median of the cases and controls was 2700 and 2500 g, respectively (*P*=0.36) (Table 2). Neither the number of the hospitalisations nor the length of the stay differed between cases and controls, but age at first hospitalisation for infection differed (Table 2). Hospitalisation for infection during the first year of life was significantly related to the development of AL (OR=2.22 (CI 95% 1.08–4.58)). Neither family history of cancer (OR=1.51 (CI 95% 0.84–2.72)) nor birth weight >2500 g (OR=1.55 (CI 95% 0.83–2.89)) showed a significant effect. When the maternal breastfeeding lasted ⩽6 months during the first months of life, the OR was 0.17 (CI 95% 0.04–0.62); when breastfeeding lasted ⩾7 months, the OR was 0.79 (CI 95% 0.25–2.46). The results of the unconditional logistical regression analysis showed an OR of 0.84 (CI 95% 0.43–1.61) for breastfeeding and AL risk, and an OR of 1.70 (CI 95% 0.82–3.52) for infections during the first year of life and the risk of AL, both of which were not significant. However, the risk of developing AL was greater if the child had been hospitalised during the first year of life, with an OR of 3.57 (CI 95% 1.59–8.05) on this model, adjusted for control variables (Table 3). The results were similar when we analysed only children with ALL (Table 3).

In subsequent analyses of children with ALL, the study population was divided according to the average age of onset of the leukaemia into ⩽6 years (⩽72 months) and >6 years (>72 months), and also by socio-economic level (low and high). Breastfeeding for ⩽6 months of life was protective for AL but not for a longer period, or, as in the case for the children from low socio-economic background, even seemed as a risk factor (Table 4). In this analyses, hospitalisation during the first year of life remained as a risk factor for children aged >6 years.

## Discussion

This is the first study in Mexico City of breastfeeding and infections in relation to the development of AL in children with DS. We found that children who had been breastfed ⩽6 months had a lower risk of developing AL, independent of their socio-economic level. In the general analysis, we found that, although early infections overall were not related to AL, those during the first year, that were severe enough to warrant hospitalisation were associated with increased risk of AL. This was notably higher for children >6 years of age than for those ⩽6 years.

The measurement of infections during the first year of life using a questionnaire, may introduce errors, given that the considerable interval since the events had occurred and the interview of the mother. The median age between cases and controls was 8.5 and almost 7 years, respectively; for this the precision of recalling the infections during the first year or the duration of breastfeeding is low, but this situation was similar among the two populations. Therefore, proxy measurements of infective exposures (such as the number of children with whom they live, or nursery school attendance) have been recommended during this period of life (McNally and Eden, 2004). The medical records of each patient provide the best assessment of infection during the first year of life (Roman *et al*, 2007). In this study, we asked about infections during the first year of life, and whether hospitalisation had been required. The fact that, in almost 80% of the cases, the parents could name the pathology for which the child was hospitalised, gave us a certain degree of confidence in the information supplied.

The size of the sample studied is smaller than those reported in other studies in children with DS; however, it should be mentioned that (1) this study consisted of 100% of the cases of children diagnosed with DS and AL under 19 years of age, who were listed in the Registry of AL in Mexico City during the study period and (2) in this period, the number of cases with DS by year in only one city was considerably greater than that reported in national registries (Canfield *et al*, 2004; Linabery *et al*, 2006).

The protective effect of breastfeeding has been observed principally for children of 5 years or younger, the age when the peak incidence of AL occurs (Kwan *et al*, 2004) and when breastfeeding has been proposed to have an immuno-modulating effect (Kirsten and Morgan, 2008). We cannot explain why a protective effect should only be evident for breastfeeding for 6 months of life or less, and not with longer periods, but it is relevant that the effect is not statistically significant.

The finding that hospitalisations for infections during the first year of life was a risk factor for ALL in children older than 6 years with DS is a new finding. However, it is consistent with the report by Roman *et al* (2007) that the infection during the first year of life was a risk factor for ALL at ages 2–5 years, particularly when the infection occurred during the first month of life. It may be relevant that a study in Mexico City reported a double incidence peak for AL, at 2–3 and 6–9 years of age (Bernaldez-Rios *et al*, 2008). If infections during the first year of life are a genuine factor, our findings may imply that in Mexico City these peaks have different etiologies with infection being more important for the 6–9 years age group. The relevant agents may be viewed as having an indirect leukemogenic effect, as *in situations* reported in relation to cancer in adults (Matsumoto *et al*, 2008).

That infectious agents are associated with the development of AL in Mexico City is supported by the fact that (1) the incidence of ALL is higher than in most developed countries, (2) infections are also more prevalent and (3) lymphomas, which could also be associated with infectious agents, have an earlier age of onset among children in Mexico City than reported for developed countries (Mejia-Arangure *et al*, 2005b). Although these arguments must be assessed in other studies, our results do not support the Greaves's hypothesis that early infection is protective against childhood ALL.

## Figures and Tables

**Table 1:** Descriptive analysis of the variables for children with Down syndrome, residing in Mexico City (1998–2006), included in this study

	**Cases (*N***=**57)**		**Controls (*N***=**218)**		
**Variables**	***N***	**%**	***N***	**%**	**OR (CI 95%)**
*Type of leukaemia*					
ALL	45	79.2	NA	NA	NA
AML	12	20.8	NA	NA	NA
Maternal breastfeeding	36	63.2	142	65.1	0.92 (0.50–1.69)
Family history of cancer	33	57.9	104	47.7	1.51 (0.84–2.72)
Male	27	47.4	117	53.7	0.78 (0.43–1.39)
Firstborn child	8	14.0	73	33.5	0.32 (0.15–0.72)
Child's weight at birth >2500 g	39	68.4	127	58.3	1.55 (0.83–2.89)
Infection during child's first year of life	16	28.1	56	25.7	1.13 (0.59–2.17)
					
*Hospitalisation for infection during child's first year of life*	14	24.5	29	13.3	2.22 (1.08–4.58)
Gastrointestinal illness	3	20.0	7	24.1	0.943^*^
Respiratory tract infection	9	60.0	16	55.2	
Other	3	20.0	6	20.7	
Presence of allergies in child	17	29.8	75	34.4	0.81 (0.43–1.52)
Low standard of living	45	78.9	153	70.2	1.59 (0.79–3.21)
Cardiovascular illness in child	7	12.3	57	26.1	0.39 (0.17–0.92)
Age of mother at delivery >35 years	22	38.6	76	34.9	1.17 (0.64–2.14)
Smoking by father during wife's pregnancy	32	56.1	128	58.7	0.90 (0.50–1.62)
Smoking by mother during pregnancy	15	26.3	63	28.9	0.88 (0.45–1.70)
Alcohol consumption by father before pregnancy	52	91.2	187	85.8	1.72 (0.64–4.65)
Alcohol consumption by mother during pregnancy	30	52.6	164	75.2	0.36 (0.20–0.67)

ALL=acute lymphoblastic leukaemia; AML=acute myeloblastic leukaemia; CI=confidence intervals; NA=not applicable; OR=odds ratio.

^*^P-value.

**Table 2:** Descriptive analysis of the continuous variables for children with Down syndrome, residing in Mexico City (1998–2006), included in this study

	**Cases (*N***=**57)**		**Controls (*N***=**218)**		
**Variable**	**Median**	**Minimum-maximum**	**Median**	**Minimum-maximum**	***P*-value** a
Maternal breastfeeding (in months)	5.69	0–36	3.77	0–48	0.78
Age of child (in months) at the time of diagnosis^b^, or at time of interview of parents^c^	106	1–227	82	1–227	0.04
Child's weight at birth (g)	2700	1200–4000	2500	900–4100	0.36
Age of mother at delivery	32	16–43	33	15–46	0.47
Number of hospitalisation by infections in child	1.00	1–3	1.00	1–12	0.88
Time (in days) for hospitalisation by infections in child	5	1–20	7	2–30	0.35
Age at hospitalisation by infection in child (months)	7	4.03	4	3.79	0.03^d^

a Mann–Whitney *U*-test.

b Cases.

c Controls.

d Student's *t*-test.

**Table 3:** Logistic regression model for maternal breastfeeding and early infections in children with Down syndrome in the whole population of the study and in children just with acute lymphoblastic leukaemia

	**Whole population with AL**		**Children with ALL**	
**Variables**	**OR** a	**CI 95%**	**OR** a	**CI 95%**
Maternal breastfeeding	0.84	0.43–1.61	0.74	0.36–1.49
Breastfeeding ⩽6 months	0.49	0.23–1.04	0.49	0.21–1.11
Breastfeeding ⩾7 months	1.45	0.67–3.11	1.33	0.57–3.09
Hospitalisation for infection during the child's first year of life	3.57	1.59–8.05	3.45	1.37–8.66
Infection during the child's first year of life	1.70	0.82–3.52	1.45	0.64–3.30

AL=acute leukaemia; ALL=acute lymphoblastic leukaemia; CI=confidence intervals; OR=odds ratio.

a The model was adjusted for the following variables: sex, weight at birth <2500 g, age of child, firstborn child, low standard of living and cardiovascular diseases.

**Table 4:** Logistic regression model for maternal breastfeeding and early infections for children with Down syndrome by age at the time of diagnosis of acute lymphoblastic leukemia and by low socio-economic level

	**All the children**				**Low socio-economic level**			
	**⩽72 months of age**		>**72 months of age**		**⩽72 months of age**		>**72 months of age**	
**Variables**	**OR** a	**CI 95%**	**OR** a	**CI 95%**	**OR** b	**CI 95%**	**OR** b	**CI 95%**
Maternal breastfeeding	0.37	0.10–1.33	0.93	0.38–2.30	0.50	0.12–2.17	1.76	0.54–5.73
Breastfeeding **⩽**6 months	0.17	0.03–0.88	0.67	0.24–1.85	0.19	0.30–1.26	0.90	0.23–3.48
Breastfeeding ⩾7 months	0.81	0.19–3.44	1.68	0.54–5.21	1.24	0.24–6.55	5.71	1.30–25.05
Hospitalisation for infection during the first year of life	2.09	0.40–10.78	7.58	2.09–27.54	2.29	0.40–13.23	8.20	1.55–43.26
Infection during the first year of life	0.80	0.18–3.60	3.18	1.01–9.97	0.70	0.14–3.47	5.12	1.05–24.89

CI=confidence intervals; OR=odds ratio.

a The model was adjusted for the following variables: sex, weight at birth <2500 g, age of child, firstborn child, low standard of living and cardiovascular diseases.

b The model was adjusted for the same variables as model one, but the variable, ‘socio-economic level’, was excluded.

## References

[bib1] Bernaldez-Rios R, Ortega-Alvarez MC, Perez-Saldivar ML, Alatoma-Medina NE, Del Campo-Martinez Mde L, Rodriguez-Zepeda Mdel C, Montero-Ponce I, Franco-Ornelas S, Fernandez-Castillo G, Nuñez-Villegas NN, Taboada-Flores MA, Flores-Lujano J, Argüelles-Sanchez ME, Juarez-Ocaña S, Fajardo-Gutierrez A, Mejia-Arangure JM (2008) The age incidence of childhood B-cell precursor acute lymphoblastic leukemia in Mexico City. J Pediatr Hematol Oncol 30: 199–203 18376281 10.1097/MPH.0b013e318162bcdc

[bib2] Bronfman M, Guiscafré H, Castro V, Castro R, Gutiérez G (1988) II. La medición de la desigualdad: una estrategia metodológica, análisis de las características socioeconómicas de la muestra. Arch Invest Med (Mex) 19: 351–360 3245751

[bib3] Canfield KN, Spector LG, Robison LL, Lazovich D, Roesler M, Olshan AF, Smith FO, Heerema NA, Barnard DR, Blair CK, Ross JA (2004) Childhood and maternal infections and risk of acute leukaemia in children with Down syndrome: a report from the Children's Oncology Group. Br J Cancer 91: 1866–1872 15520821 10.1038/sj.bjc.6602223 PMC2409774

[bib4] Curado MP, Edwards B, Shin HR, Storm H, Ferlay J, Heanue M (eds) (2007) Cancer incidence in five continents. In IARC Scientific Publications Vol. IX, publication No. 160 The International Agency for Research on Cancer (IARC): Lyon, France

[bib5] Greaves M (2005) In utero origins of childhood leukaemia. Early Hum Dev 81: 123–129 15707724 10.1016/j.earlhumdev.2004.10.004

[bib6] Greaves M (2006) Infection, immune responses and the aetiology of childhood leukaemia. Nat Rev Cancer 6: 193–203 16467884 10.1038/nrc1816

[bib7] Hasle H, Clemmensen IH, Mikkelsen M (2000) Risks of leukaemia and solid tumours in individuals with Down's syndrome. Lancet 355: 165–169 10675114 10.1016/S0140-6736(99)05264-2

[bib8] Kirsten E, Morgan A (2008) Does infection cause or prevent childhood leukaemia. A review of the scientific evidence. Children with leukaemia. http://www.leukaemia.org/what-we-do/fund-research/prevention-project. Accessed on 6 October 2008.

[bib9] Kwan ML, Buffler PA, Abrams B, Kiley VA (2004) Breastfeeding patterns and risk of childhood acute lymphoblastic leukaemia: a meta-analysis. Public Health Rep 119: 521–535 15504444 10.1016/j.phr.2004.09.002 PMC1497668

[bib10] Linabery AM, Olshan AF, Gamis AS, Smith FO, Heerema NA, Blair CK, Ross JA, Children's Oncology Group (2006) Exposure to medical test irradiation and acute leukemia among children with Down syndrome: a report from the Children's Oncology Group. Pediatrics 118: 1499–1508 10.1542/peds.2006-0644 17030598

[bib11] MacArthur AC, McBride ML, Spinelli JJ, Tamaro S, Gallagher RP, Theriault GP (2008) Risk of childhood leukemia associated with vaccination infection medication use in childhood: the cross-Canada childhood leukemia study. Am J Epidemiol 167: 598–606 18079130 10.1093/aje/kwm339

[bib12] Matsumoto S, Yamasaki K, Tsuji K, Shirahama S (2008) Human T lymphotropic virus type 1 infection and gastric cancer development in Japan. J Infect Dis 198: 10–15 18544011 10.1086/588733

[bib13] McNally RJ, Eden TO (2004) An infectious aetiology for childhood acute leukaemia: a review of the evidence. Br J Haematol 127: 243–263 15491284 10.1111/j.1365-2141.2004.05166.x

[bib14] Mejia-Arangure JM, Bonilla M, Lorenzana R, Juarez-Ocana S, de Reyes G, Perez-Saldivar ML, Gonzalez-Miranda G, Bernaldez-Rios R, Ortiz-Hernandez A, Ortega-Alvarez M, Martinez-García Mdel C, Fajardo-Gutiérrez A (2005a) Incidence of leukemias in children from El Salvador and Mexico City between 1996 and 2000: population-based data. BMC Cancer 5: 33 15807901 10.1186/1471-2407-5-33 PMC1090561

[bib15] Mejia-Arangure JM, Flores Aguilar H, Juarez Muñoz I, Vazquez Langle J, Games Eternod J, Perez Saldivar ML, Ortega Alvarez MC, Rendon Macias ME, Gutierrez AF (2005b) Age of onset of different malignant tumors in childhood. Rev Med Inst Mex Seguro Soc 43: 25–37 15998478

[bib16] Menegaux F, Olshan AF, Neglia JP, Pollock BH, Bondy ML (2004) Day care, childhood infections, and risk of neuroblastoma. Am J Epidemiol 159: 843–851 15105177 10.1093/aje/kwh111 PMC2080646

[bib17] National Cancer Institute, Division of cancer epidemiology and genetics (1998)Questionnaire modules (The QMOD website). Available at: http://dceg.cancer.gov/QMOD/

[bib18] O'Connor SM, Boneva RS (2007) Infectious etiologies of childhood leukemia: plausibility and challenges to proof. Environ Health Perspect 115: 146–150 17366835 10.1289/ehp.9024 PMC1817664

[bib19] Robison LL (1992) Down syndrome and leukemia. Leukemia 6(Suppl 1): 5–7 1532221

[bib20] Roman E, Simpson J, Ansell P, Kinsey S, Mitchell CD, McKinney PA, Birch JM, Greaves M, Eden T (2007) United Kingdom Childhood Cancer Study Investigators. Childhood acute lymphoblastic leukemia and infections in the first year of life: a report from the United Kingdom Childhood Cancer Study. Am J Epidemiol 165: 496–504 17182983 10.1093/aje/kwk039

[bib21] Ross JA, Spector LG, Robison LL, Olshan AF (2005) Epidemiology of leukemia in children with Down syndrome. Pediatr Blood Cancer 44: 8–12 15390275 10.1002/pbc.20165

